# Identification of MicroRNAs as Potential Biomarker for Gastric Cancer by System Biological Analysis

**DOI:** 10.1155/2014/901428

**Published:** 2014-05-28

**Authors:** Wenying Yan, Shouli Wang, Zhandong Sun, Yuxin Lin, Shengwei Sun, Jiajia Chen, Weichang Chen

**Affiliations:** ^1^Department of Gastroenterology, The First Affiliated Hospital of Soochow University, Suzhou 215006, China; ^2^Taicang Center for Translational Bioinformatics Center for Systems Biology, Taicang 215400, China; ^3^Department of Pathology, Soochow University School of Medicine, Suzhou 215123, China; ^4^Center for Systems Biology, Soochow University, No. 1 Shizi Street, Suzhou 215006, China; ^5^School of Chemistry, Biology and Material Engineering, Suzhou University of Science and Technology, Suzhou 215011, China

## Abstract

Gastric cancers (GC) have the high morbidity and mortality rates worldwide and there is a need to identify sufficiently sensitive biomarkers for GC. MicroRNAs (miRNAs) could be promising potential biomarkers for GC diagnosis. We employed a systematic and integrative bioinformatics framework to identify GC-related microRNAs from the public microRNA and mRNA expression dataset generated by RNA-seq technology. The performance of the 17 candidate miRNAs was evaluated by hierarchal clustering, ROC analysis, and literature mining. Fourteen have been found to be associated with GC and three microRNAs (miR-211, let-7b, and miR-708) were for the first time reported to associate with GC and may be used for diagnostic biomarkers for GC.

## 1. Introduction

Gastric cancer (GC) or stomach cancer (SC), the fourth leading cancer worldwide, is a biologically heterogeneous disease. It is the second major contributor to mortality caused by cancer [1, 2]. GC is most common in the Asian and Pacific Islanders and the incidence and death rate are more than twice those in Whites [3]. The occurrence and development of GC is multiple step and multiple factorial processes. The risk factors for gastric cancer include* Helicobacter pylori* infection, advanced age, diet low in fruits and vegetables or high in salted, smoked, preserved foods, chronic atrophic gastritis, and family history of gastric cancer [4–7].

MicroRNAs are small, single-stranded, and noncoding RNAs that negatively regulate gene expression at the posttranscriptional level [8]. Multiple studies have shown differential expression of microRNAs between cancer and normal tissues. Aberrant changes in microRNAs expression have been shown to be associated with lung cancers [9], breast cancers [10], prostate cancers [11], and others. MicroRNAs are therefore the promising candidates as diagnostic, prognostic, and predictive biomarkers in cancers. Various studies have investigated important role of the microRNAs in gastric cancers [12–17].

**Figure 1:**
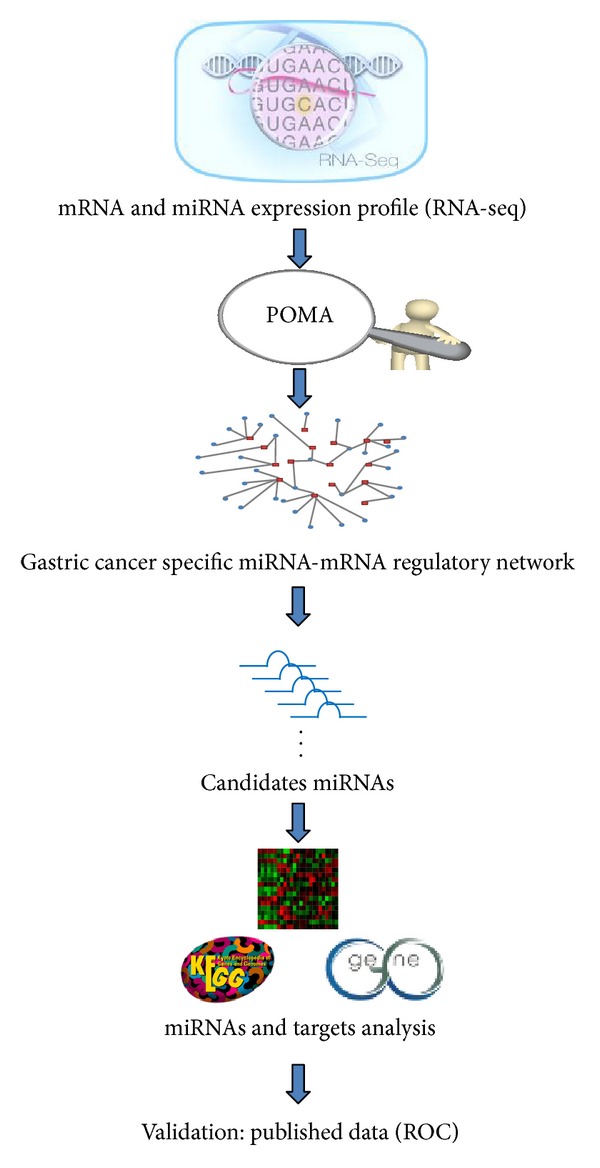
Analysis pipeline in this study.

However, gastric cancers are systems biology diseases and the heterogeneity and complexity of carcinogenesis complicate the marker identification process. Herein we employed an integrated systems biology approach to identifying candidate miRNAs as biomarkers that could differentiate patient with gastric cancer from healthy controls. The analysis pipeline of this paper is shown in Figure 1.

## 2. Methods

### 2.1. Dataset Collection and Outlier Differential Expressed Genes Detection

We explored expression profiles (GSE36968 from NCBI GEO) for gastric cancer (GC) and noncancerous gastric tissue samples [18]. The dataset was generated by the AB SOLiD System 3.0 (Homo sapiens). The dataset includes 30 transcriptomic profiles, 6 from noncancerous gastric tissues and 24 from gastric tumor samples. Among the 30 samples, 25 of them have paired miRNA and mRNA expression profiles. These 25 samples, which contain 6 noncancerous gastric tissue samples and 19 gastric tumor samples, were selected for further analysis. The clinical information of the samples was summarized in Table 1 and the detailed information was listed in Additional file 1 in Supplementary Material available online at http://dx.doi.org/10.1155/2014/901428. We directly downloaded the processed expression data and used log transformation of the expression values for the following analysis.

Outlier microRNAs and genes were detected with least sum of ordered subset square *t*-statistic (LSOSS) [19] and implemented in R scripts by Karrila et al. [20]. We used the spearman correlation method to detect negative correlations between outlier miRNAs and outlier genes. The cutoff for correlation coefficient was chosen to be −0.6 and *P* value < 0.05. Thus we got the significant inverse expression pattern specific for the gastric cancer.

### 2.2. Refinement of Candidate Gastric Cancer MicroRNAs with the Pipeline of Outlier MicroRNA Analysis (POMA)

We employed an in-house prediction model POMA to identify the candidate GC miRNAs from the outlier miRNAs detected by LSOSS. POMA is an integrative method to identify candidate cancer miRNA biomarkers from the miRNA regulatory network by linking paired miRNA and gene expression data and highly reliable miRNA-mRNA interaction data [21]. The main hypothesis of POMA is that if the deregulated genes are targeted exclusively by certain miRNA, this very miRNA is more likely to show regulatory activity. Based on the in-depth exploration of miRNA regulatory network, we conclude that miRNAs with greater independent regulatory power were more likely to be potential biomarkers in human. POMA has successfully identified miRNAs as potential biomarkers in prostate cancer [21], clear cell renal cell carcinoma [22], and sepsis [23].

Using POMA, we mapped the inverse expression pattern of miRNAs and targets to a human miRNA-mRNA interaction network to construct a GC-specific miRNA-mRNA interaction subnetwork. The human miRNA-mRNA interaction network was reconstructed by a comprehensive search of experimentally validated interactions extracted from 4 databases (miRecords, miRTarbase, miR2Disease, and TarBase) and computational prediction from HOCTAR, starBase, and ExprTargetDB.

Then the Z-score was calculated to measure the probability of miRNA having regulatory role in cancer. Z-score was the ratio of number of genes targeted exclusively by a specific miRNA (*α*) and number of all the genes targeted by that miRNA (*β*). The Z-score was calculated for each miRNA in the GC-specific miRNA-mRNA interaction subnetwork. Using thresholds 0.3 of Z-score and significantly larger *α* (*α* > 1, *P* value < 0.05), we identified candidate miRNAs with potential regulatory role in GC.

### 2.3. Evaluation of the Performance of MicroRNAs

We employed the heat map and ROC analysis to evaluate the quality of candidate miRNA as GC biomarkers. Heat map and hierarchical clustering were performed by the R package “gplots” [24]. Receiver-operating characteristic (ROC) curves were constructed and the area under ROC curves (AUC) was calculated to evaluate the performance of each candidate miRNA. The parameters sensitivity, specificity, and accuracy were also provided for the miRNAs.

### 2.4. Functional Enrichment of MicroRNA Targets

The target genes of candidate miRNAs were mapped to different databases, including gene ontology (GO), KEGG, and MetaCore pathway maps and diseases (by Biomarkers) ontology for functional enrichment analysis. GO and KEGG pathway enrichment were performed using Database for Annotation, Visualization and Integrated Discovery (DAVID) [25, 26]. MetaCore pathway maps and diseases (by Biomarkers) ontology enrichment analysis were performed by MetaCore from Thomson Reuters. MetaCore calculates the *P* value by hypergeometric distribution to evaluate the statistical significance of the enriched pathways and diseases (by Biomarkers) and uses false discovery rate adjustment for multiple test correction.

## 3. Results

### 3.1. Detection of Gastric Cancer Specific miRNA-mRNA Subnetwork

**Figure 2:**
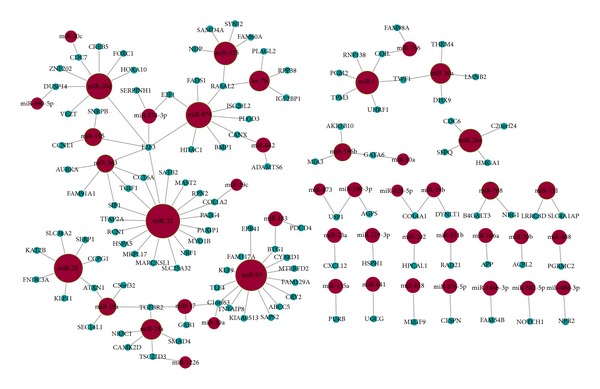
Gastric cancer specific miRNA-mRNA subnetwork. Red nodes and blue nodes denote miRNAs and target genes, respectively. miRNAs nodes with green border are candidate miRNAs as biomarkers.

Using POMA, we mapped the significant inverse expression pattern from the gastric cancer data to the human miRNA-mRNA interaction network to get the GC specific miRNA-mRNA subnetwork as illustrated in Figure 2 and the edge list of the network was shown in Additional file 2. The subnetwork contains 161 nodes, 46 miRNAs (red nodes), 115 target genes (green nodes), and 142 interactions.

### 3.2. Identifying Candidate miRNA as Biomarkers for GC

**Figure 3:**
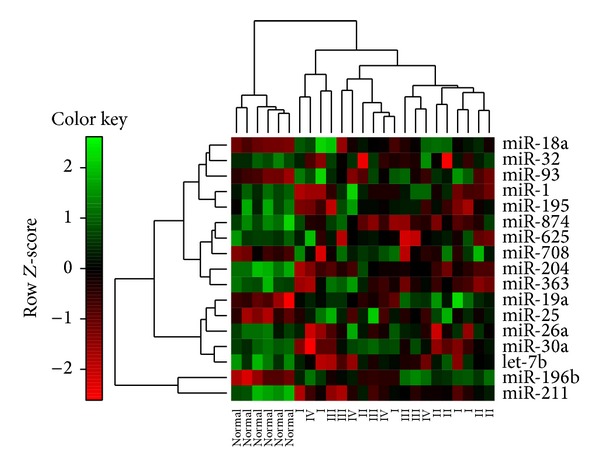
Hierarchical clustering of 19 cancer samples and 6 normal samples with the 17 candidate miRNAs. Every row represents individual miRNA, and each column represents individual sample.

**Figure 4:**
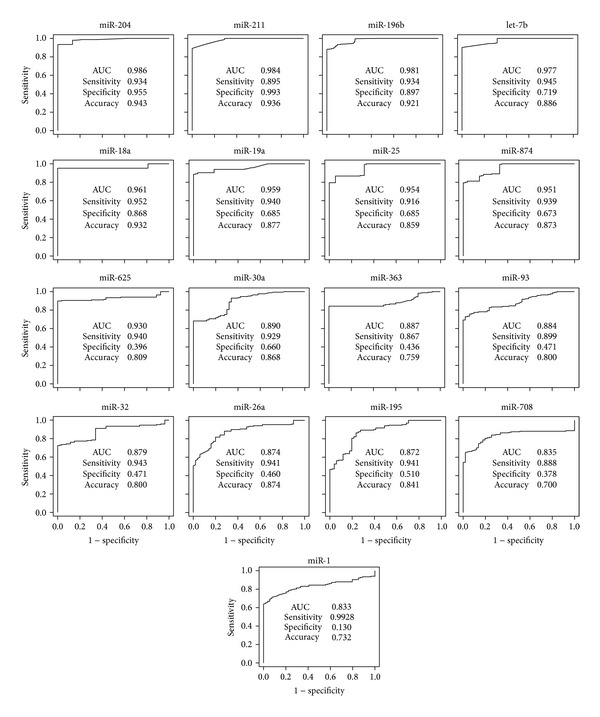
ROC curve of candidate GC miRNAs. AUC: area under the curve.

We retrieved a set of seventeen candidate miRNAs (Table 2) through POMA and evaluated their performance as biomarkers in three aspects. First, using hierarchical clustering based on the candidate miRNAs expression, we successfully separated the 19 samples of cancer tissue and 6 normal samples into discrete groups (Figure 3). The ROC curves for the 17 candidate miRNAs are presented in Figure 4. The areas under the ROC curve (AUC) for the 17 miRNAs are 0.833–0.986, and overall accuracy is 73.2%–94.3%. The identified miRNAs are able to effectively differentiate patients with the GC from controls.

Furthermore, we also performed the literature search of the seventeen miRNAs to validate their role in the regulation of GC; see Table 2. Fourteen of them have been reported for their roles in gastric cancer by the low-throughput experiment, such as miR-204 targets Bcl-2 [27] and SIRT1 [28] with downregulation in gastric cancer. Although the remaining four miRNAs were not proved in low-throughput experiment, they have their role in gastric cancer or other cancers. miRNA-30a is ensembled with other six miRNAs as a seven-miRNA signature which is closely associated with relapse-free and overall survival among patients with gastric cancer [29]. miRNA-211 has contribution to colorectal cancer cell growth [30], melanoma cell invasion [31], and head and neck carcinomas [32]. The expression level of miR-708 reflects differences between colorectal carcinogenesis and normal samples [33] and it may play an important role as a tumor suppressor in human glioblastoma cells [34]. let-7b was upregulated in the acute myeloid leukemia when compared to healthy controls [35]. let-7b in GC patients with low HMGA2 (high mobility group A2) expression was significantly higher than in those with high HMGA2 expression and high expression of HMGA2 in GC correlates was an independent prognostic factor [36]. Therefore, the four miRNAs may be the potential biomarkers for gastric cancer.

### 3.3. Function Enrichment of Candidate miRNAs Target Genes

**Figure 5:**
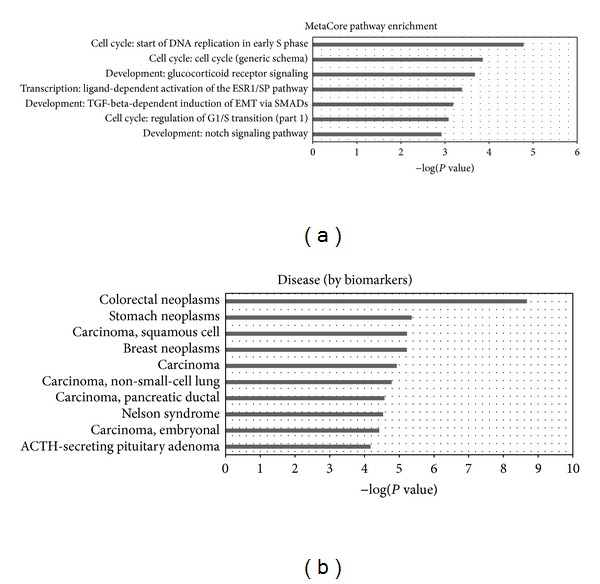
Functional enrichment analysis of target genes. (a) is the significantly enriched MetaCore pathway map. (b) is the significantly enriched disease (biomarkers) ontology.

The candidate miRNAs, along with their regulated genes, provide potential miRNA-mRNA target pairs in gastric cancer. The targets of these miRNAs were mapped to functional databases, including GO, KEGG, and MetaCore (Figure 5 and Additional file 3). The significantly enriched GO terms (*P* value < 0.05 and FDR < 0.05) include regulation of RNA metabolic process, regulation of transcription from RNA polymerase II promoter, regulation of transcription, DNA-templated and regulation of transcription. KEGG pathways that are significantly enriched with the candidate miRNAs targets are associated with cancer, for example, cell cycle, pancreatic cancer, pathways in cancer, and prostate cancer.

The enriched (*P* value < 0.05 and FDR < 0.05) MetaCore pathway maps converge on cell cycle, development, and transcription, as shown in Figure 5 and Table 3. Then we searched the PubMed for published papers describing their constituent network objects in GC to evaluate the relevance of these pathway maps in gastric cancer. All of the enriched pathways have at least ten objects related to gastric cancer; see Additional file 4.

Disease (biomarkers) ontology in MetaCore is created based on the classification in Medical Subject Headings (MeSH), a controlled vocabulary of medical terms created by the National Library of Medicine (http://www.nlm.nih.gov). Each disease in diseases ontology has its corresponding biomarker gene or a set of genes. The stomach neoplasms disease term ranked top three among the enriched diseases. There are 41 objects in the stomach neoplasms that were mapped by the candidate miRNAs target genes. All these results further confirmed the correlation between these target genes and GC and, hence, testified the reliability of our predicted miRNAs as gastric cancer biomarkers.

## 4. Discussion

In this study, we identified 17 miRNAs using a systematic and integrative method POMA from RNA-seq based expression profile. We first applied LSOSS to detect differentially expressed microRNAs and genes from the RNA-seq data. LSOSS generally outperforms the *t*-statistics and is more competent for cancer data analysis, as our previous studies indicated [22, 37]. Then the inverse expression pattern of miRNAs and genes was predicted by the spearman correlation.

Using POMA, we got gastric cancer specific miRNA-mRNA subnetwork and 17 candidate GC miRNAs for biomarkers with regulatory roles. The performance of the identified miRNAs was evaluated by hierarchical clustering and ROC curve. Moreover, literature mining confirmed that 14 out of the 17 candidate miRNAs have been reported to have aberrant expression in GC, which lends credibility to our finding. The remaining three miRNAs, miR-211, miR-708, and let-7b, have no previous annotation in GC, but their role in other digestive systems cancers has been reported. miR-211 expression promotes colorectal cancer cell growth [30] and could be a prognostic factor in resected pancreatic ductal adenocarcinoma [38]. miR-708 undergoes aberrant expression in colorectal carcinogenesis samples [33] and pancreatic intraepithelial neoplasias samples [39]. In colorectal liver metastases, invasion front-specific downregulation of let-7b plays a pivotal role in tumor progression [40]. let-7 (let-7b and let-7c) expression has relationship with response to chemotherapy in patients with esophageal cancer and can be potentially used to predict the response to cisplatin-based chemotherapy in esophageal cancer [41]. To our best knowledge, this is the first report that the three microRNAs (miR-211, miR-708, and let-7b) could be the candidate biomarkers for human gastric cancers.

Functional enrichment analysis of the candidate miRNAs target genes revealed some important biological process and pathway maps. Most GO biological process terms are about the regulation processes, for example, the regulation of RNA metabolic process and regulation of transcription. The enriched GO terms in molecular function were also associated with transcription activity such as microRNA regulation activity. The GO enrichment results agree well with the regulatory concepts of microRNAs. MicroRNAs repress their target genes to fine-tune distinct gene regulatory programs. In cancer, microRNAs play either oncogenic or tumor suppressive role. Oncogenic microRNAs downregulate tumor suppressor genes directly, whereas tumor suppressor microRNAs might lead to the upregulation of oncogenes. In this way, microRNAs regulate cancer progression and dictate specific disease phenotypes. It is also observed that microRNAs are tightly related to other families of regulators, such as transcription factors in gene regulatory networks. They work synergistically to regulate gene expression. So it is not surprising that the targets of GC-related microRNAs converge in gene regulatory processes.

KEGG pathways that are significantly enriched with candidate miRNA targets were all associated with cancers, for example, chronic myeloid leukemia, pancreatic cancer, pathways in cancer, and prostate cancer. It is worth noting that the enriched pathways from both KEGG and MetaCore are involved in cell cycle. For example, in the MetaCore, the top two significantly enriched pathways: the start of DNA replication in early S phase and cell cycle (generic schema) belong to the cell cycle category. The remaining pathways also have important roles in gastric cancer, such as the famous TGF-beta signaling pathway [42–44]. We further evaluated the relevance of the enriched MetaCore pathway maps to gastric cancer by performing the text mining at the objects levels in each pathway and found that all these pathways contain at least ten critical genes in gastric cancers.

According to the disease ontology enrichment analysis, the stomach neoplasm was the second most enriched disease ontology in MetaCore pathways, colorectal neoplasm being the top enriched one. The reason may be that the colorectal neoplasms category incorporates more genes (8014 genes) than stomach neoplasms (3101 genes) in MetaCore database. Thus genes are more likely to be enriched in the colorectal neoplasms. Additionally, colorectal neoplasms and stomach neoplasms share some genes.

The experimental validation is a necessary task to be done after the identification of putative gastric cancer related miRNAs. This is our research plan for the future. Since we did not verify the miRNAs directly in this study, we provided some “indirect evidences” to validate our result by text mining. Although not perfect, text mining helps us to mine previously discovered differential miRNAs and pathways from large volumes of literature, which can help reduce the number of our predicted cancer associated pathways, and to expedite the biological validation of the pathways of interest.

Because the main goal of this research is to identify viable biomarkers of GC diagnosis, we only grouped the samples into 2 major categories: cancer versus noncancerous. Such a binary classification has not fully considered the clinical aspects of each sample. As a future perspective, patients could be subdivided into well-defined small groups according to their unique clinical features, for example, stage, histologic, and therapeutic response. In this manner, the individual difference of cancer mechanism is accounted. This kind of investigation will help to find population-specific biomarkers and facilitate personalized diagnosis, prognosis, or treatment of gastric cancer.

In conclusion, we identified 17 microRNAs that are associated with gastric cancers and 3 of them (miR-211, let-7b, and miR-708) could be potential novel biomarkers for gastric cancer diagnosis and treatment. The candidates predicted herein need further wet-lab validation.

## Supplementary Material

Additional File 1: Clinical information for 25 samples, including sex, age, GC type and histologic type.
Additional File 2: GC Specific miRNA-mRNA Network.
Additional File 3: Enriched Functional Themes for Target Genes of Candidate miRNAs.
Additional File 4: Enriched MetaCore Pathways and Constituent GC-Related Objects.

## Figures and Tables

**Table 1:** Clinical information of 25 samples.

Characteristic		Sample (*n* = 25)
Age	Median	66
	Range	32–83

Sex	Male	20
	Female	5

Stage	Stage I	5
	Stage II	5
	Stage III	5
	Stage IV	4
	Normal	6

Histology	Mixed	2
	Diffuse	9
	Intestine	5
	Unknown	3
	Normal	6

**Table 2:** Aberrantly expressed miRNAs in gastric cancer detected by low-throughput methods.

miRNA	Expression in GC	Detection technology	Study design	PMID
miR-204	Down	RT-PCR/QRT-PCR RT-PCR QRT-PCR	Cell lines Tissue Tissue	23768087 23152059 21416062
miR-211	—	—	—	—
miR-196b	Up	QRT-PCR	Tissue	21416062 24222951
let-7b	—	—	—	—
miR-18a	Up	QRT-PCR	Tissue	21671476
miR-19a	Up	QRT-PCR	Tissue Cell lines	23621248
miR-25	Up	Northern blotting	Tissue	19153141
miR-874	Down	QRT-PCR	Cell lines	23800944
miR-625	Down	QRT-PCR	Tissue	22677169
miR-30a	—	—	—	—
miR-363	Up	QRT-PCR	Cell lines	23975832
miR-93	Up	QRT-PCR	Tissue	18328430
miR-32	Up	QRT-PCR	Tissue	21874264
miR-26a	Down	QRT-PCR	Tissues Cell lines	24015269
miR-195	Down	RT-PCR	Tissue	21987613
miR-708	—	—	—	—
miR-1	Up	QRT-PCR	Serum	21112772

**Table 3:** The significant GeneGo pathway maps enriched with candidate miRNAs target genes.

Pathway maps	Pathway map category	Ration of mapped targets	*P* value	PubMed citation number
Start of DNA replication in early S phase	Cell cycle	4/32	1.650*E −* 05	37
Cell cycle (generic schema)	Cell cycle	3/21	1.387*E −* 04	75
Glucocorticoid receptor signaling	Development	3/24	2.089*E −* 04	76
Ligand-dependent activation of the ESR1/SP pathway	Transcription	3/30	4.105*E −* 04	319
TGF-beta-dependent induction of EMT via SMADs	Development	3/35	6.505*E −* 04	292
Regulation of G1/S transition (part 1)	Cell cycle	3/38	8.298*E −* 04	238
Notch signaling pathway	Development	3/43	1.193*E −* 03	29
