# Supplementary material for: Identification of MicroRNAs as Potential Biomarker for Gastric Cancer by System Biological Analysis
Source: Biomed Res Int. 2014 May 28;2014:901428. doi: 10.1155/2014/901428 (PMC4058523; doi:10.1155/2014/901428)
Supplement: Supplementary file 1 — Additional File 1: Clinical information for 25 samples, including sex, age, GC type and histologic type. Additional File 2: GC Specific miRNA-mRNA Network. Additional File 3: Enriched Functional Themes for Target Genes of Candidate miRNAs. Additional File 4: Enriched MetaCore Pathways and Constituent GC-Related Objects. [file 901428.f1.pdf]

Clinical information for each sample

GSM No.	Sex	Age	Type	Histologics
GSM907559	M	74	stage Ⅳ	Diffuse
GSM907569	F	60	stage Ⅳ	Mixed
GSM907570	M	69	stage Ⅳ	Diffuse
GSM907581	M	unknown	stage Ⅳ	unknown
GSM907560	M	63	stage Ⅲ	Intestine
GSM907576	M	47	stage Ⅲ	Intestine
GSM907577	M	61	stage Ⅲ	Intestine
GSM907579	M	55	stage Ⅲ	Diffuse
GSM907580	M	72	stage Ⅲ	Diffuse
GSM907561	F	70	stage Ⅱ	Diffuse
GSM907566	M	69	stage Ⅱ	Diffuse
GSM907571	M	52	stage Ⅱ	unknown
GSM907574	M	66	stage Ⅱ	Intestine
GSM907582	M	unknown	stage Ⅱ	unknown
GSM907558	M	83	stage Ⅰ	Mixed
GSM907562	M	76	stage Ⅰ	Diffuse
GSM907564	M	61	stage Ⅰ	Intestine
GSM907573	F	66	stage Ⅰ	Diffuse
GSM907578	M	64	stage Ⅰ	Diffuse
GSM907563	F	67	Normal	Normal
GSM907565	M	69	Normal	Normal
GSM907567	M	37	Normal	Normal
GSM907568	M	56	Normal	Normal
GSM907572	F	32	Normal	Normal
GSM907575	M	66	Normal	Normal

Ref: Kim, Y.H., et al., AMPKalpha modulation in cancer progression: multilayer integrative analysis of th[illegible]

[illegible] whole transcriptome in Asian gastric cancer. Cancer Res, 2012. 72(10): p. 2512-21.
