# Supplementary material for: Identification of MicroRNAs as Potential Biomarker for Gastric Cancer by System Biological Analysis
Source: Biomed Res Int. 2014 May 28;2014:901428. doi: 10.1155/2014/901428 (PMC4058523; doi:10.1155/2014/901428)
Supplement: Supplementary file 2 [file 901428.f2.pdf]

GC Specific miRNA-mRNA Network

miRNA	Gene
let-7b	IGF2BP1
let-7b	RASAL2
let-7b	RPP38
let-7b	PLAGL2
miR-1	RNF138
miR-1	PGM2
miR-1	COIL
miR-1	TPM3
miR-1	UHRF1
miR-1	TWF1
miR-106a	APP
miR-10a	GATA6
miR-1226	TSC22D3
miR-129-3p	HSPH1
miR-135a	PURB
miR-140-3p	AGPS
miR-140-3p	USP1
miR-146b-3p	FAM54B
miR-17	C1orf63
miR-17	GAB1
miR-17	TGFBR2
miR-183	BTG1
miR-183	PDCD4
miR-18a	CAMK2D
miR-18a	NR3C1
miR-18a	SMAD4
miR-18a	TGFBR2
miR-18a	TSC22D3
miR-195	SNRPB
miR-195	CCNE1
miR-195	E2F3
miR-196b	GATA6
miR-196b	MIA3
miR-196b	AKR1B10
miR-19a	C5orf32
miR-19a	SEC14L1
miR-19a	ATXN1
miR-19a	TGFBR2
miR-202	HPCAL1
miR-204	DUSP14
miR-204	VEZT
miR-204	CDC7

miR-204	CREB5
miR-204	E2F3
miR-204	FOXC1
miR-204	ZNF202
miR-204	HOXA10
miR-20a	C1orf63
miR-20b	ACPL2
miR-211	SLC4A1AP
miR-211	LRRC8D
miR-23a	CXCL12
miR-25	CCPG1
miR-25	ATXN1
miR-25	SLC38A2
miR-25	KLF11
miR-25	FNDC3A
miR-25	SERP1
miR-25	KAT2B
miR-26a	C20orf24
miR-26a	SFPQ
miR-26a	HMGA1
miR-26a	CDC6
miR-29b	DYNLT1
miR-29b	COL4A1
miR-29c	COL1A2
miR-30a	DHX9
miR-30a	LMNB2
miR-30a	THEM4
miR-30a	TWF1
miR-30c	CDC7
miR-32	RPN2
miR-32	MARCKSL1
miR-32	E2F3
miR-32	SIP1
miR-32	MYO1B
miR-32	MAST2
miR-32	NRF1
miR-32	TFAP2A
miR-32	COL1A2
miR-32	TGIF1
miR-32	CCT6A
miR-32	RCN1
miR-32	PA2G4
miR-32	PAXIP1
miR-32	HSPA5

miR-32	MRPL17
miR-32	SLC25A32
miR-32	SATB2
miR-363	TGIF1
miR-363	E2F3
miR-363	CCT6A
miR-363	AURKA
miR-363	FAM91A1
miR-363	SIP1
miR-486-3p	NPR2
miR-488	PGRMC2
miR-490-5p	DUSP14
miR-551b	RAD21
miR-574-3p	E2F3
miR-574-3p	SERPINH1
miR-574-3p	E2F1
miR-582-5p	NOTCH1
miR-618	MEGF9
miR-625	SAMD4A
miR-625	SYNJ2
miR-625	RASAL2
miR-625	NDP
miR-625	FAM60A
miR-628-5p	COL4A1
miR-641	UGCG
miR-642	CANX
miR-642	ADAMTS6
miR-708	B4GALT3
miR-708	NRG1
miR-766	COIL
miR-766	FAM98A
miR-873	USP1
miR-874	ISG20L2
miR-874	FADS1
miR-874	PLOD3
miR-874	HDAC1
miR-874	E2F1
miR-874	BMP1
miR-874	RASAL2
miR-874	E2F3
miR-874	CANX
miR-876-5p	CLSPN
miR-93	TLE4
miR-93	EPB41

miR-93	KLF9
miR-93	MTERFD2
miR-93	BTG1
miR-93	KIAA0513
miR-93	SAPS2
miR-93	C1orf63
miR-93	CRY2
miR-93	FAM129A
miR-93	FAM117A
miR-93	ABCC5
miR-93	CYBRD1
miR-93	TNFAIP8
