# Supplementary material for: Identification of MicroRNAs as Potential Biomarker for Gastric Cancer by System Biological Analysis
Source: Biomed Res Int. 2014 May 28;2014:901428. doi: 10.1155/2014/901428 (PMC4058523; doi:10.1155/2014/901428)
Supplement: Supplementary file 3 [file 901428.f3.pdf]

Enriched Functional Themes for Target Genes of Candidate miRNAs

**GO_Biology process (pvalue<0.05, FDR<0.05)**	**GO_Molecular function (pvalue<0.05, FDR<0.05)**
GO:0051252~regulation of RNA metabolic process	GO:0030528~transcription regulator activity
GO:0006357~regulation of transcription from RNA polymerase II promoter	GO:0003700~transcription factor activity
GO:0006355~regulation of transcription, DNA-dependent	
GO:0045449~regulation of transcription	

**GO_Cellular component (pvalue<0.05, FDR<0.05)**	**KEGG (pvalue<0.05)**
GO:0031981~nuclear lumen	hsa04110:Cell cycle
GO:0070013~intracellular organelle lumen	hsa05220:Chronic myeloid leukemia
GO:0043233~organelle lumen	hsa05212:Pancreatic cancer
GO:0031974~membrane-enclosed lumen	hsa05200:Pathways in cancer
GO:0005654~nucleoplasm	hsa05215:Prostate cancer
GO:0044451~nucleoplasm part	

**MetaCore Pathway Maps**
Start of DNA replication in early S phase
Cell cycle (generic schema)
Glucocorticoid receptor signaling
Ligand-dependent activation of the ESR1/SP pathway
TGF-beta-dependent induction of EMT via SMADs
Regulation of G1/S transition (part 1)
Notch Signaling Pathway
