# Supplementary material for: Identification of MicroRNAs as Potential Biomarker for Gastric Cancer by System Biological Analysis
Source: Biomed Res Int. 2014 May 28;2014:901428. doi: 10.1155/2014/901428 (PMC4058523; doi:10.1155/2014/901428)
Supplement: Supplementary file 4 [file 901428.f4.pdf]

Enriched MetaCore P

idate miRNAs Target Genes Enriched MetaCore Pat	Pubmed citiation Number	GC related Genes in the pathway
Cell cycle_Start of DNA replication in early S phase	37	CCNE1
		CDC6
		CDK2
		E2F1
		MCM2
		MCM4
		MCM5
		MCM7
		ORC1
		RPA3
Cell cycle_Cell cycle (generic schema)	75	CCNA2
		CCNB1
		CCND1
		CCND2
		CCND3
		CCNE1
		CDC25A
		CDC25B
		CDC25C
		CDK1
		CDK2
		CDK4
		CDK6
		E2F1
		E2F2
		E2F3
		RB1
		RBL2
Glucocorticoid receptor signaling	76	CEBPB
		FOS
		HSP90AA1
		HSP90AB1
		HSP90B1
		HSPA14
		HSPA1A
		HSPA1B
		HSPA1L
		HSPA4
		HSPA5
		HSPA8
		JUN
		MMP13
		NFKB1
		NFKB2
		NFKBIA
		NR3C1

		POU2F1
		POU2F2
		PTGES3
		RELA
		SERPINE1
		SMAD3
		STAT5A
		STAT5B
		TGFBR1
Ligand-dependent activation of the ESR1/SP pathway	319	ACTB
		ADA
		CAD
		CCND1
		CCNE1
		CDC25A
		CEBPB
		E2F1
		EGFR
		ESR1
		FOS
		HIF1A
		LDLR
		NCOA3
		SMARCA2
		SMARCA4
		SMARCB1
		SP1
		TYMS
		VEGFA
		CDH1
		CDH2
		CLDN1

TGF-beta-dependent induction of EMT via SMADs	292	ETS1
		FN1
		HMGA2
		ILK
		JAG1
		MMP2
		NOTCH1
		OCLN
		SERPINE1
		SMAD2
		SMAD3
		SMAD4
		SNAI1
		SNAI2
		SP1
		TCF3
		TGFB1
		TGFB2
		TGFB3
		TGFBR1
		TGFBR2
		TWIST1
		VIM
		ZEB1
		ZEB2
Regulation of G1/S transition (part 1)	238	ANAPC1
		BRCA1
		BTRC
		CCNA2
		CCND1
		CCND2
		CCND3
		CCNE1
		CDC25A
		CDK2
		CDK4
		CDK6
		CDKN1A
		CDKN1B
		CDKN2A
		CDKN2B
		CHEK2
		CUL1
		FBXW7

		GSK3B
		PPP2R3A
		RPS6KB1
		SKP2
		SMAD2
		SMAD3
		SMAD4
		SP1
		TGFB1
		TGFB2
		TGFBR1
		TGFBR2
		UBB
		UBC
Notch Signaling Pathway	29	CUL1
		FBXW7
		H3F3A
		H3F3B
		HDAC1
		HDAC2
		HES1
		HIST1H4A
		HIST1H4B
		HIST1H4C
		HIST1H4D
		HIST1H4E
		HIST1H4F
		HIST1H4H
		HIST1H4I
		HIST1H4J
		HIST1H4K
		HIST1H4L
		HIST2H4A
		HIST2H4B
		HIST3H3
		HIST4H4
		JAG1
		JAG2
		MFNG
		MYOD1
		NFKBIA
		NOTCH1
		RBBP4
		TP73

Pathways and Constituent GC-Related Objects

PMID
10218458,10547573,10666388,11002215,11038303,11059220,12064799,12133540,12493027,12667381,12799636,15746045,16270527,16575401,18841391,19058005,19096232,19569046,20965966,7559076,9242335,9529014,9811181
17151793,17229543
10666388,12667381,17366642
10225440,15701848,
11782383,18373176,20694513,20965966
20965966
20694513
20965966
20965966
17229543
20334896,20525401
12200597,15484292,19724852,20334896,21777278
10218458,10404130,10492044,10547573,10547574,10666388,10672060,11059220,11559529,12064799,12133540,14691913,14966904,15378768,16163549,16270527,16490596,17393422,17447856,18184402,18224443,18484097,18609126,18841391,19084934,19096232,19160099,19760977,19822419,20334896,20397048,20525401,21275459,215598
10547574,10666388,12064799,12133540,12771922,9778110
12064799,12133540
10218458,10547573,10666388,11002215,11038303,11059220,12064799,12133540,12493027,12667381,12799636,15746045,16270527,16575401,18841391,19058005,19096232,19569046,20965966,7559076,9242335,9529014,9811181
17934831,9414655
11782383,15273739,15701848,17934831,20965966,9414655
9414655
14654553,18841391,20965966
10666388,12667381,17366642
10666388,11782383,18484097
19167610,23442884
10225440,15701848,
20965966
10225440
12508351,12973852,14691913,15818729,15906946,17131485,17652530,18484097,18996365,19058005,20205286,20
16998811
12809678,15701848,16247479,16474375,16981245
18104429,18158562,18570890,20682178
18723843,19203381,19569046,20530692
15273739,18723843,19203381
18482745
20965966
12957361,18723843,19626584
12957361,18478330,18723843,19626584
19626584
10705241,12579213,17927838
16718772,17187227,18482745,18723843,21382870
18723843
11821786,16474375,18570890
12673424,14604892,19562509
14714615,1683872,16949912,17429689,18835621,19231580,19509001,21575498,22776619,23002344,23117246
14966904
20132559,21376060
18510611,19569046,21409489

16278805
11782383
11903579
11751513,14716817,15014040,15154625,15682491,15814628,16949912,18184402,19865524,20478305,20946117,23
10340041,12708473,16435385,16630137,17972143,19594546,20549826,8187075,9006092,9041148
14647420
19175826,22328012
22328012
10873359,12692838,18174246,19958625
18723843
15701848
11782383,20965966
10218458,10404130,10492044,10547573,10547574,10666388,10672060,11059220,11559529,12064799,12133540,14691913,14966904,15378768,16163549,16270527,16490596,17393422,17447856,18184402,18224443,18484097,18609126,18841391,19084934,19096232,19160099,19760977,19822419,20334896,20397048,20525401,21275459,215598
10218458,10547573,10666388,11002215,11038303,11059220,12064799,12133540,12493027,12667381,12799636,15746045,16270527,16575401,18841391,19058005,19096232,19569046,20965966,7559076,9242335,9529014,9811181
17934831,9414655
12809678,15701848,16247479,16474375,16981245
10225440,15701848,
10037277,11680933,12460468,12579211,12679307,1282800,1317752,14991403,15017643,15073595,15647181,16816379,17094480,17431415,17516110,17896140,17922051,18199332,18224443,18397279,18483367,18544998,18835621,18957060,19061514,19142899,19173907,19259093,19479974,19949675,20368841,20430735,20530692,20937558,
11804739,12439914,12679906,15897574,16294196,17046193,17652530,18510611,18829507,19399343,20339947,20
18104429,18158562,18570890,20682178
16557436,16847924,16951228,17094412,17166265,17179985,17461470,17984117,18025805,18452596,19287200,19
12162407
10861496,15701848,17229543
18006815
18006815
22490415
1282800,14695137,15747170,15818728,16035620,16158248,17085651,17473187,19296249,19563076
12530000,12975018,15244514,15284183,15316940,15386366,15640503,15837757,15930032,16045580,16317430,16
10026754,10070308,10357403,10379858,10437615,10440683,10461065,10526273,10779624,10854553,10878459,10965312,11061344,11197049,11223822,11244997,11506954,11579392,11819218,11819817,11957070,11978184,12131083,12173384,12174362,12378339,12433614,12453275,12460468,12493028,12509950,12647634,12668286,12708473,12767510,12811205,12825129,12854133,12854174,12901801,14532971,14669355,14710231,15029262,15069709,15112337,15112366,15116331,15153787,15274366,15309707,15312686,15457582,15515423,15569052,15746037,15864753,16004820,16086118,16115916,16124061,16179839,16495697,16557436,16761623,16810742,16847924,16948814,16998811,17085650,17094412,17094486,17096728,17111394,17143338,17171787,17224647,17262742,17342999,17409493,17426061,17461470,17520388,17633783,17671399,17687613,17896140,17922682,17972143,17984117,18023552,18025805,18095080,18181047,18184402,18200665,18234057,18273500,18317966,18373164,18383582,18452596,18506690,18516318,18609713,18972599,19173829,19173907,19287200,19296249,19419943,19496079,1956
10094558,10211998,10231415,10461060,10604738,10664629,10749913,11106237,11113859,11194192,11313896,11564215,11598162,11665847,11684825,11705864,11706763,11782383,11783066,11856141,11920500,12060614,12080224,12121238,12209998,12210063,12216071,12414534,12439911,12444556,12448005,12460468,12520583,12548420,12558744,12631658,12692837,12702045,12750281,12923325,12940439,12966349,1333788,14580691,14729585,14742272,14750169,14998854,15102685,15102690,15128072,1514246,15198362,15330201,15361706,15500652,15522555,15609397,15870832,15918205,16127748,16132582,16215948,16219136,16237750,16244541,16356709,16474375,16610016,16821608,16929514,16969501,17066436,17139167,17201188,17224647,17299729,17380028,17512904,17545690,17549573,17589897,17634543,17652530,17671701,17786966,17852867,17883295,17908458,17914577,18095267,18197935,18312357,18342503,18377425,18386788,18395897,18482459,18569997,18768510,18825309,1882
11564215,12414534,17512904,19107131,19309396,23752181
15842637,16112005,16220299,20462599

11098083,14562368,14604892,8952528
11782383,14973074,15273739,15330800,16430945,17512904,17978572,19107131,21365014
17229543,18413822
12596061
19491270,22691042
10999748,11819490,11823976,11903578,12460468,14604892,14973074,15522165,15929171,16124061,16538217,16718824,16731602,16940985,17094486,17153464,17236757,17908458,18330957,18498066,19309396,19383080,19562509,19569980,19586554,19822020,20028745,20478305,20563765,20730428,21148629,21337552,21526499,216124
19491270,21466361,22691042
12667324
10340041,12708473,16435385,16630137,17972143,19594546,20549826,8187075,9006092,9041148
19351925
14647420
11234879,12136244,15033661,15501972,17200344,17634543,19341727,21105199,9331080
12414534,19309396,22018628,23670240,23752181
17299729,19107131,21274735,23752181
1282800,14695137,15747170,15818728,16035620,16158248,17085651,17473187,19296249,19563076
17299729
10080606,10526273,10839293,11234879,11821786,12353499,12794244,12809678,14669355,16310402,16509870,17096728,17187359,17262742,17270215,17562261,17640324,18341540,18484655,18687755,18936038,19550422,1956
17270215,19550422,21777278,9139831
17270215,19550422,9139831
10873359,12692838,18174246,19958625
10398102,10873359,12353499,17096728,17187359,17562261
12414534,17297478,17512904,17886095,19107131,20005068,20405297,20737169,21104359
14973074,19107131,19309396,22018628,22189617
22018628
12414534
18622497
11196187,18829507,19399343,20205286,20530692,20965966
17295679
20334896,20525401,21858804
10218458,10404130,10492044,10547573,10547574,10666388,10672060,11059220,11559529,12064799,12133540,14691913,14966904,15378768,16163549,16270527,16490596,17393422,17447856,18184402,18224443,18484097,18609126,18841391,19084934,19096232,19160099,19760977,19822419,20334896,20397048,20525401,21275459,215598
10547574,10666388,12064799,12133540,12771922,9778110
12064799,12133540
10218458,10547573,10666388,11002215,11038303,11059220,12064799,12133540,12493027,12667381,12799636,15746045,16270527,16575401,18841391,19058005,19096232,19569046,20965966,7559076,9242335,9529014,9811181
17934831,9414655
10666388,12667381,17366642
10666388,11782383,18484097
19167610,23442884
10218458,10547573,10695995,10718178,11197049,11463497,11521990,11745255,11903579,11932915,12417790,12794244,12872447,14647439,15036662,15069711,15586362,16121348,16289646,17166265,18497953,18751407,1905
10218458,10223564,10404130,10666388,10692028,10779641,11002215,11038303,11042561,11147595,11358008,11903579,12097295,12239454,12451619,14654553,15645119,16121348,16480585,18841391,19096232,20334896,2155
10705239,10779641,10830779,10861481,11146446,12060614,12194996,12210063,12448005,12460459,12514789,12608655,12632489,12692837,12717828,12824886,12890389,12894891,12973852,14691913,15240535,15330201,15352040,15459500,15498792,15526354,15612883,15818729,15859515,16474375,16518809,16534497,16619489,16821608,17058198,17074152,17139167,17460447,17652530,17671701,17914577,17971901,18395906,18484097,18622497,
12060614,12448005,17652530,19399343,23504555
17685927
21190721
16824748,17909001,19366810

16371352,17160944,20447678,20704706,20854473
20965966
20003385
12097295,14654553,15645119,16425372,20965966
19351925
14647420
11234879,12136244,15033661,15501972,17200344,17634543,19341727,21105199,9331080
1282800,14695137,15747170,15818728,16035620,16158248,17085651,17473187,19296249,19563076
10080606,10526273,10839293,11234879,11821786,12353499,12794244,12809678,14669355,16310402,16509870,17<br>096728,17187359,17262742,17270215,17562261,17640324,18341540,18484655,18687755,18936038,19550422,1956
17270215,19550422,21777278,9139831
10873359,12692838,18174246,19958625
10398102,10873359,12353499,17096728,17187359,17562261
11294777,23013936
23013936
21190721
16824748,17909001,19366810
23065444
23065444
11749695,14731134,18207460
15865607
21466361,22691042
11782383,20965966
11782383,20965966
11782383,20965966
11782383,20965966
11782383,20965966
11782383,20965966
11782383,20965966
11782383,20965966
11782383,20965966
11782383,20965966
11782383,20965966
11782383,20965966
11782383,20965966
19896696
11782383,20965966
19491270,22691042
22691042
21092330
20664982
20132559,21376060
19491270,21466361,22691042
11821786
10815895,12430182,17058198,17094900,18829507,19386249,19399343,20530692,20737169
